# Engagement in perinatal depression treatment: a qualitative study of barriers across and within racial/ethnic groups

**DOI:** 10.1186/s12884-021-03969-1

**Published:** 2021-07-16

**Authors:** Esti Iturralde, Crystal A. Hsiao, Linda Nkemere, Ai Kubo, Stacy A. Sterling, Tracy Flanagan, Lyndsay A. Avalos

**Affiliations:** 1 Division of Research, Kaiser Permanente Northern California, 2000 Broadway, Oakland, CA 94612 USA; 2 The Permanente Medical Group, Oakland, CA USA

**Keywords:** Perinatal depression, Racial disparities, Health equity, Behavioral health integration, COM-B

## Abstract

**Background:**

To better understand previously observed racial/ethnic disparities in perinatal depression treatment rates we examined care engagement factors across and within race/ethnicity.

**Methods:**

Obstetric patients and women’s health clinician experts from a large healthcare system participated in this qualitative study. We conducted focus groups with 30 pregnant or postpartum women of Asian, Black, Latina, and White race/ethnicity with positive depression screens. Nine clinician experts in perinatal depression (obstetric, mental health, and primary care providers) were interviewed. A semi-structured format elicited treatment barriers, cultural factors, and helpful strategies. Discussion transcripts were coded using a general inductive approach with themes mapped to the Capability-Opportunity-Motivation-Behavior (COM-B) theoretical framework.

**Results:**

Treatment barriers included social stigma, difficulties recognizing one’s own depression, low understanding of treatment options, and lack of time for treatment. Distinct factors emerged for non-White women including culturally specific messages discouraging treatment, low social support, trauma history, and difficulty taking time off from work for treatment. Clinician factors included knowledge and skill handling perinatal depression, cultural competencies, and language barriers. Participants recommended better integration of mental health treatment with obstetric care, greater treatment convenience (e.g., telemedicine), and programmatic attention to cultural factors and social determinants of health.

**Conclusions:**

Women from diverse backgrounds with perinatal depression encounter individual-level, social, and clinician-related barriers to treatment engagement, necessitating care strategies that reduce stigma, offer convenience, and attend to cultural and economic factors. Our findings suggest the importance of intervention and policy approaches effecting change at multiple levels to increase perinatal depression treatment engagement.

## Background

Perinatal depression affects 12–20% of pregnant and postpartum women [1, 2] and is associated with severe health consequences for mother and child [3–9]. Professional medical societies recommend routine perinatal depression screening and follow-up treatment as key components of care [10–12]. Despite an increase in perinatal depression identification in healthcare settings [13, 14], treatment engagement remains lower among non-White versus White women [15–19].

Past qualitative studies have examined potential barriers to perinatal depression treatment engagement, including low mental healthcare access [20–26]. However, there is a need to understand barriers across and within racial/ethnic groups that may persist despite access. We examined factors associated with perinatal depression treatment engagement by race/ethnicity among insured (public and private) patients in a healthcare system with equal access to depression care and other services. Drawing from patient focus groups and clinician expert interviews, we synthesized treatment engagement factors, comparing across race/ethnicity. We also report participants’ recommendations for how to increase women’s engagement in depression care. To generate brief, practical summaries addressing these areas, we used a general inductive analytic approach and mapped findings onto a theoretical framework (the Capability-Opportunity-Motivation Behavior [COM-B] System [27]), which helps to link insights to intervention and policy objectives.

## Methods

### Study setting

Participants were patients or clinicians in Kaiser Permanente Northern California (KPNC), a large integrated healthcare system with medical facilities in 14 counties caring for > 44,000 pregnancies annually. KPNC members (30% of the regional population) are enrolled through employer-based plans, Medicare, Medicaid, and health insurance exchanges. KPNC obstetric care (OBGYN) routinely screens patients using the Patient Health Questionnaire (PHQ-9) [28] during perinatal care [13, 29]. Mental healthcare is a covered benefit for KPNC members. OBGYN physicians initiate depression care through: referral to a perinatal depression psychotherapy group; prescription of antidepressant medications; referral to an OBGYN-based behavioral health provider; or referral to KPNC specialty mental health services for individual/group psychotherapy or antidepressant medication management.

### Patient focus groups

Eligible patients were Asian, Black, Latina, or White pregnant and postpartum (up to 12 months) adult women with significant perinatal depressive symptoms (PHQ-9 score ≥ 10) per electronic health records (EHR) who were contacted from a random sample of eligible participants drawn from the Northern California region. The PHQ-9 is widely used for perinatal depression screening and has been validated across diverse clinical settings and cultural contexts [30]. By telephone, study staff consented patients, assessed depressive symptoms and treatment history, and scheduled focus group participation (2 groups per race/ethnicity, 8 total, 2–7 participants per group). A clinical psychologist (EI) experienced in conducting focus groups by telephone moderated the 1-h telephone focus groups using a semi-structured guide exploring perinatal depression experiences, cultural factors, the role of OBGYN, and treatment preferences. Telephone was selected for focus groups to overcome geographic, convenience-, and stigma-related barriers to participation, increasing the representativeness of the sample [31].

### Clinician interviews

KPNC clinicians with a professional focus in women’s health and perinatal depression were identified through KPNC perinatal depression clinical champion professional networks and verbally consented by telephone. EI moderated 30-min telephone interviews using a semi-structured guide assessing factors influencing patient follow-through from perinatal depression screening to treatment, the role of race/ethnicity, and opinions about how to improve depression treatment engagement.

### Qualitative analysis

Focus groups and interviews were conducted July to September 2018. We analyzed audio transcripts using a general inductive approach [32] during February to August 2019. A consultation group of five women researchers from different racial/ethnic backgrounds (Asian/Japanese [AK], Asian/Chinese-American [CH], White/Spanish-American [EI], Black/Nigerian-American [LN], White/American [LA]) read transcripts and identified thematic categories related to treatment engagement (e.g., stigma, OBGYN provider role). EI and LA assigned quotations to these categories in NVivo qualitative analysis software version 12 [33]. Through iterative summary documents and discussions, EI and LA generated specific themes, checking conclusions with the consultation group. EI and LA mapped these themes to the COM-B model [27], a transtheoretical framework facilitating translation of research findings into clinical implementation by linking potential intervention and policy components to core behavior change mechanisms. The goal behavior (depression treatment engagement) is thus driven by factors related to capability (e.g., knowledge), motivation (decision-making or emotional processes), and opportunity (social and other environmental factors). A full analysis of the patient transcripts was followed by analysis of the clinician transcripts, and results were compared by race/ethnicity and patient/clinician role. EI and LA generated a list of recommended interventions based on examples provided by participants. The KPNC Institutional Review Board approved study procedures.

## Results

Thirty patients (10 Asian, 5 Black, 6 Latina, and 9 White women) participated (Table 1). The mean age was 34 years; 10 patients were pregnant and 20 postpartum. Patients’ depressive symptoms ranged from mild to moderate (score range = 5–19). Two-thirds of women (*n* = 21) reported receiving mental health treatment during pregnancy or postpartum. In focus groups, patients described significant perinatal depression symptoms, including inappropriate guilt and self-blame, low self-worth, feeling overwhelmed and hopeless, difficulty getting out of bed, and excessively sad affect. Some reported prior severe symptoms including paranoia and suicidal ideation.

**Table 1:** Participant characteristics

**Patient characteristic, n (%)**	**Overall (*N* = 30)**	**Asian (*n* = 10)**	**Black (*n* = 5)**	**Latina (*n* = 6)**	**White (*n* = 9)**
Age, mean years ± SD	34 ± 5	32 ± 5	33 ± 7	34 ± 6	36 ± 5
Perinatal Stage					
Pregnant	10 (33)	2 (20)	2 (40)	4 (67)	2 (22)
Postpartum	20 (67)	8 (80)	3 (60)	2 (33)	7 (78)
College graduate or more	13 (43)	6 (60)	2 (40)	1 (17)	4 (44)
Medicaid	5 (17)	0 (0)	2 (40)	2 (33)	1 (11)
Neighborhood Deprivation^a^	12 (41)	4 (40)	3 (75)	3 (50)	2 (22)
Depressive symptoms, mean ± SD PHQ-8 score	10 ± 4	10 ± 5	11 ± 7	10 ± 3	10 ± 3
Past treatment	21 (70)	6 (60)	5 (100)	4 (67)	6 (67)
Treatment type					
Psychotherapy only	8 (38)	2 (33)	2 (40)	3 (75)	1 (17)
Medication only	3 (4)	0 (0)	1 (20)	0 (0)	2 (33)
Combination	10 (48)	4 (67)	2 (40)	1 (25)	3 (50)
**Clinician characteristic (*N* = 9)**	**n**				
Specialty					
OBGYN	6				
Internal medicine	1				
Psychologist	1				
Licensed clinical social worker	1				
Race/ethnicity					
Asian	2				
Black	1				
White	6				
Years in practice, median (range)	18 (8–30)				

^a^Above-average Neighborhood Deprivation Index: a census tract-based index summarizing across socioeconomic domains, with higher scores representing more deprivation [34]

Nine clinician experts participated in interviews (6 OBGYN physicians, 1 primary care physician with expertise in perinatal depression, and 2 mental health therapists leading perinatal depression psychotherapy groups). All were women; 2 were Asian, 1 Black, and 6 White. Time in practice ranged from 8 to 30 years.

Table 2 presents depression treatment engagement factors and illustrative quotes by patient or clinician role organized by the COM-B model. We present themes corresponding to the individual-level domains (capability and motivation) first, followed by those related to the social/environmental domain (opportunity). We highlight below contrasts across race/ethnicity and between patient and clinician perspectives.

**Table 2:** Individual, social, and clinician factors

**Description**	**Patient Quotations**		**Clinician Quotations**	
**Individual Capability**				
Low recognition of treatment need	*I felt depressed during all three pregnancies…. This time… I have acknowledged the severity of it.* —Black patient		*[For many women with perinatal depression] they were so overwhelmed they didn’t even know what was going on. [By the time they seek treatment], they have a major depression, it’s recurrent, it’s pretty deep, and it’s harder to treat.* —Mental health clinician	
	*I just was in such denial…. I was in such a state that was so normal to me that I didn’t really know that it was postpartum depression.* —White patient			
Low understanding of how treatment works	*I am pretty sure I got [information about depression] in the packet when we left the hospital [after giving birth], but when you get home… you don’t really have time to think about, “oh there are all those resources I can use.”* —White patient		*[Patients need to know] that group [treatment] is effective and not just [the healthcare system’s strategy] to limit how much mental healthcare they can have.* —Mental health clinician	
	*[When I was depressed] it was just four weeks of torture, of just, “When can I be seen? When can I be seen?” because I couldn’t figure out who I was supposed to call and how to even get a referral [for depression treatment].* —White patient		*There’s not great advertising about [the facility’s postpartum depression group]. Unless someone tells them about it, they really don’t know.* —OBGYN physician	
**Individual Motivation**				
Attitudes towards antidepressant medication	*[During my pregnancy] I ended up needing a low dose of [an antidepressant medication]. My new provider felt that my anxiety was just really, really bad, and it would be more harmful to the pregnancy than the medication.* —Asian patient		*I always offer [antidepressant medication] to them, and very few [women with perinatal depression] will take it.* —OBGYN physician	
	*I am extremely open to going on medication [after I give birth], because I would love to do anything that I can in order not to have postpartum depression again…. For now, I’d seek therapy out to avoid the medication.* —Latina patient		*There’s this idea that [depression] is not a real illness…. [I] tell them, “If… you had to take this medication to bring down your blood pressure to help the pregnancy, I don’t think you would feel as resistant as you do right now.”* —OBGYN physician	
Attitudes towards group therapy	*Being able to attend a support group and meet other moms who are experiencing the same thing, it was really helpful knowing that I wasn’t alone.* —Asian patient		*I think most patients, regardless of presenting issue, have some reluctance about group… [but] some women are going to have even more reluctance to being in group and it’s not just because of group. It’s because of culture reasons.* —Mental health clinician	
	*I wanted everything to be perfect in my world, and I didn't want anybody to know…. I didn’t want to go in front of a [depression therapy] group and say that [I had difficulties].* —Black patient			
**Social Environment**				
Culturally based stigma	*“You have to stay strong,” [people say]…. Whatever emotions you have, you push them away because you don’t want to seem weak. And just pray about it.* —Black patient		*[There are barriers to treatment engagement] related to stigma, shame, and so on. And also, related to their community’s judgment about them if they seek mental health services. —*Primary care physician	
	*If you go to the psychologist or something, it’s not well seen. It’s like you’re, oh, like a crazy person.* —Latina patient			
	*The kind of social media… perfect mommy scenario where you feel like it’s normal to get everything done…. And to admit that it does not come as easily, there’s a little bit of shame in society*. —White patient			
Fear of discrimination	*I wasn’t comfortable with accepting medication because I didn’t want it to be in my medical record…. I didn’t know if it can affect if I want to increase my life insurance or employment factors or my [professional] licensure. —*Black patient		*I have had a couple of patients where they thought they were being [racially] profiled [during behavioral health screening].* —OBGYN physician	
	*I wasn’t sure if [discussing your depression with a doctor would] affect your visa or your stay here [in the U.S.]. And I didn’t want to mess that up… so I didn’t know what to do.* —Latina patient		*For our African-American patients, there’s so much history [of discrimination] that makes all of this deeply complicated for them to get care and trust in the system. —*OBGYN physician	
Family attitudes towards treatment	*My daughter’s grandmother [would say], “Oh, you need to just pull up your bootstraps and, you know, get through it.”* —Asian patient		*A lot of times, the women themselves are very onboard [for treatment] but they’re very afraid of what their mom, mother-in-law, husband, whatever, is going to think of it. —*OBGYN physician	
	*My mom… was pretty adamant about the fact that I should go and see [a therapist].* —Black patient			
Low social support & trauma history	*I got to the point in my pregnancy that I left [my child’s] dad… because we got into it so bad that I honestly thought I was going to have a miscarriage…. And I thought, "Hmm, me and the baby or it’s you.* —Black patient		*There’s times where I think [about a patient], “This woman is really struggling with poverty. Her partner vanished. She is on WIC. She has next to no family support, and she has had addiction issues in the past.* —Mental health clinician	
	*You know, I came [to the U.S.] and my [partner] was really abusive. Then when I had my baby … I find out he was cheating…. And now that I have my baby boy, I think it reminded me of all that*. —Latina patient			
Time and resources	*We want to feel better, too, but… if it’s going to cause too much trouble in our lives to get that seven-month-old a babysitter… you have to choose what’s best for your baby. And if that means you miss that [depression treatment] appointment, then that means you miss the appointment.* —White patient		*Young Hispanic or African-American women were often in jobs where taking time off [for depression treatment] would have been a significant issue. —*Primary care physician	
**Clinical Encounter**				
OBGYN physician’s approach	*[My OBGYN provider] was focused on my medical complications more, but I didn’t feel supported with my depression with her at all*. —Black patient		*I feel confident about using [antidepressant] medications and talking with [patients] about that. But, I probably didn’t coming right out of residency…. When I went to residency, I don’t think we even talked about postpartum depression. —*OBGYN physician	
	*My first OBGYN treated me like a statistic, a number…. “Here, take this [antidepressant] medication and go away…." But my second OB has been so great. She listens, she is very understanding, and she makes me feel the communication is open. –*Latina patient			
Mental health clinician’s approach	*I really, really like who I see [OBGYN behavioral health provider]. I feel like she can relate to what I’m going through and stuff.* —Asian patient		*There are a couple [mental health providers who] are really passionate about [perinatal depression] and a couple that are possibly not as confident. —*OBGYN physician	
	*[The mental health provider] mansplained things to me, and he just assumed it was extremely bad PMS all the time…. I never went back to him after that.* —Latina patient			
Cultural (mis)under-standings	*[My therapist] was a child of immigrant parents, and I really felt connected with her because of that…. She wasn’t necessarily my same cultural background, but just being the child of immigrant parents made a huge difference because I felt like she understood where I was straddling both worlds all the time.* —Latina patient		*In some [cultural] backgrounds, it’s just not okay to be depressed. So, I try to use different language, particularly with my Latina patients. They don’t really talk about depression as much as they talk about feeling anxious or feeling down or feeling sad. That tends to work better.* —OBGYN physician	
Language barriers	*When you have a different language, I think you feel more comfortable when you have somebody who can speak your language.* —Latina patient		*It’s awkward when the interpreter [telephone service] comes on. Unfortunately, when it’s a man, I find that [patients] tend to shut down and not want to talk about some of their other problems.* —OBGYN physician	

### Capability

#### Women may not recognize treatment needs or understand treatment processes

Patients and clinicians observed that depressive symptoms often worsened to a severe level before many women became aware that they needed treatment. Severe depressive symptoms—on top of the normal challenges of pregnancy or caring for a newborn—made it more difficult for women to initiate treatment.

Women varied in their understanding of the steps necessary to treat their own depression. Among patients with a mental health history, many reinitiated care with past mental health providers; they understood treatment options and referral processes. However, for women new to mental health treatment it was often unclear how to pursue help. Clinicians worried that women did not follow through with treatment recommendations due to limited understanding of options.

### Motivation

#### Attitudes towards antidepressant medication and group psychotherapy may influence treatment engagement

OBGYN physicians reported frequent patient refusal of antidepressant medications (or failure to pick up prescriptions) due to stigma or feared side effects. However, patients across race/ethnicity offered nuanced views of medications, including past or current medication treatment during or after pregnancy. Some women wished to avoid medications (e.g., due to potential fetal health concerns; to avoid dampening emotions; because antidepressant medications were seen as over-prescribed). Others expressed openness to medications if psychotherapy was not effective.

Clinicians viewed perinatal depression groups as effective but noted widespread patient reluctance to attend sessions due to stigma and fears about self-disclosure. Clinicians observed that patients who overcame reluctance were often surprised by the benefits of groups. One mental health clinician noted that non-White and low-income women needed prior reassurance that groups would feel comfortable to women of many backgrounds. Clinicians also recommended framing the group as a “class” to reduce stigma.

Patients expressed a range of opinions about psychotherapy groups. Several women reported past positive experiences prior to their pregnancies. However, others viewed group psychotherapy negatively (e.g., self-disclosure to the group caused them anxiety; it did not provide sufficient individual attention; group psychotherapy was mainly a cost-cutting measure for the healthcare system). Some patients viewed perinatal depression-specific groups in a similar negative light. However, many other patients appreciated unique advantages of perinatal groups (e.g., offering social support from women with similar challenges; providing openness to women bringing infants to groups).

Many women preferred meeting individually with a psychotherapist, but often encountered structural hurdles in the healthcare system (e.g., limited therapist availability; limited opportunity to switch therapists). Some higher income women in the Asian and White focus groups reported paying out of pocket to see a psychiatrist or psychotherapist outside of their health plan.

### Opportunity

#### Stigma or discrimination may affect treatment engagement

Patients described stigmatizing beliefs in their families and communities about perinatal depression, with nuances based on race/ethnicity. Women in all groups observed cultural beliefs that perinatal depression reflected a personal deficit or failure as a mother. Some Asian patients described familial pressures to hide depressive symptoms. Some Black patients shared cultural beliefs that depression signified weakness and could be overcome by prayer. Some Latina patients voiced concern that their depression made them seem ungrateful for motherhood or “crazy.” Some White and Asian patients described shame induced by social media portraying “perfect mommies” who seemed depression-free.

Some clinicians viewed family disapproval of treatment as particularly influential for Asian and Latina patients. They viewed some Asian patients as hiding depressive symptoms from clinicians due to family preferences towards privacy. They reported that Latina patients declined treatment due to family expectations that they were responsible for all parenting duties and could not take time out for treatment. One mental health clinician reported frequently educating her non-White patients that depression was not just “a White woman’s disease.”

Patients and clinicians also described ways that perinatal depression treatment could invoke fears of discrimination. Among their non-White patients, clinicians observed fears of being screened excessively due to “racial profiling” and general distrust that the majority White medical establishment would meet their health needs. Some Black and Latina patients expressed possible economic or legal consequences of having documented depression treatment, including threats to immigration status or employment.

#### Low social support and trauma history may complicate treatment engagement

Patients across race/ethnicity noted family/partner support as instrumental in their pursuing treatment, but many women did not receive this support. Non-White patients described having low social support resulting from emigration away from their extended family or dissolution of their partner relationship. Uniquely Black and Latina participants described significant life histories of trauma and poverty, which exacerbated depressive symptoms. Some Black and Latina patients also received critical support from community resources (e.g., job readiness/mentoring program; visiting nurse service for mothers; community mental health agency).

Clinicians viewed their non-White patients as more likely to face multiple social and economic stressors (“social determinants of health,” e.g., food insecurity, intimate partner violence), which complicated the process of diagnosing and treating perinatal depression. During brief routine appointments, OBGYN physicians struggled to prioritize and attend to these multiple factors. Some clinicians described working with social workers in OBGYN to address patients’ urgent social needs as a first step towards addressing perinatal depression symptoms.

#### Women may lack time and resources for treatment

Across groups, women cited time constraints as a major barrier to treatment engagement. Given infant care and other responsibilities, women found the multi-step process of engaging in treatment infeasible. Mental health visits may entail taking time off from work, traveling to the medical facility, arranging for childcare, and otherwise disrupting tight schedules (e.g., infants’ naptime). Several Black and Latina patients described strict constraints related to maternity leaves and time off from work. A clinician similarly observed that inflexible work schedules, in addition to higher co-pays or deductibles, could pose significant barriers to treatment engagement to many non-White patients in particular.

#### Women may prefer OBGYN for depression treatment

Patients recognized a vital role for OBGYN providers in showing empathy, screening for perinatal depression, monitoring symptoms, discussing treatment options, and facilitating treatment. OBGYN physicians noted perinatal depression management as a neglected topic in their formal training, and some disclosed a lack of confidence about antidepressant medication management. As opposed to referral to a general mental health clinic, patients and clinicians described a benefit to integrating mental healthcare into OBGYN clinics through the presence of embedded behavioral health providers who could conduct psychotherapy and monitor patients.

Patients and clinicians perceived various barriers inherent in treating perinatal depression in a general mental health clinic. They frequently cited a lack of awareness among general mental health providers about perinatal depression, which led providers to make dismissive remarks (described by one patient as “mansplaining”) and to other problems establishing trust with general mental health providers. Patients described quitting treatment prematurely because they struggled to find a mental health provider who understood their unique challenges. Patients viewed mental health appointments as inconvenient, requiring them to visit an unfamiliar department at the medical facility. Clinicians perceived treatment in the mental health clinic as aversive to patients because of stigma related to mental illness. OBGYN physicians also perceived difficulty coordinating care with the general mental health clinic.

#### Cultural and linguistic factors may influence treatment engagement

According to patients and clinicians, women’s engagement in depression treatment could be derailed by providers’ cultural missteps. Several clinicians expressed concern that providers could be insensitive to cultural or economic pressures faced by women, leading to unrealistic or offensive advice. Participants stated that it was important for clinicians to take time to understand patients’ perspectives, but that time constraints posed a barrier. Several non-White patients described the clinical visit as too rushed to allow for clinicians to establish trust.

Non-White patients generally dismissed the need to have a mental health provider of the same race/ethnicity, but some did recall positive experiences with psychotherapists of the same race/ethnicity or other background. Several clinicians reported concern that the lack of racial/ethnic diversity among mental health providers deterred women of color from engaging in treatment. Clinicians attempted to bridge racial/ethnic differences with their patients by learning about cultural perinatal practices, and by discussing depression with patients using less stigmatizing language (e.g., related to feelings, relationships, or physiology versus mental health).

Lack of English proficiency posed additional barriers, according to clinicians. Patients might avoid disclosing depressive symptoms in front of medical interpreters, especially men. Language interpreting also interfered with patients’ participation in perinatal depression groups. Clinicians lamented the lack of multilingual mental health clinicians and recognized a need for more educational materials about perinatal depression in different languages.

### Participants’ recommendations

Participants observed a need for *outreach, education, preventive care, and convenience* (Table 3)*.* Universal screening coupled with clinician-initiated monitoring and follow-up could relieve some of the burden of treatment initiation. Women also favored convenience, such as being able to bring children to mental health visits, or through telemedicine appointments, which since these interviews have become more prevalent in depression care due to COVID-19 stay-at-home restrictions. Clinicians suggested educational initiatives to demystify and normalize the treatment process, emphasizing that perinatal depression is a common medical complication that affects women from all backgrounds. Patients and clinicians noted the value of educating women’s partners about perinatal depression.

**Table 3:** Recommendations to increase treatment engagement

**Patient Outreach and Education**	
Prevention, Monitoring & Convenience	Normalizing Treatment
• Offer preventive education and services • Proactively monitor and follow up after depression screening • Increase convenience through telemedicine, on-site childcare	• Educate patients about depression as a medical condition that affects women from all backgrounds • Demystify treatment options • Educate family members about perinatal depression
**Community and Social Determinants of Health**	
Community Engagement	Diversity & Inclusivity
• Engage community resources, peer health workers to spread awareness • Consult with patient advisories to develop culturally appropriate messages • Monitor patients’ social concerns and refer to social work and community resources	• Increase racial/ethnic and linguistic diversity among clinicians • Create language-specific materials and treatment options
**Clinician Training and Support**	
Integrated Perinatal Depression Management	**Cultural Tailoring**
• Train OBGYN and mental health clinicians in perinatal depression • Embed a mental health provider in OBGYN who can facilitate depression treatment • Coordinate depression care among a team of providers	• Provide culturally specific training to clinical providers

Several clinicians emphasized the need to go beyond the clinic to *involve community members and resources* in spreading perinatal depression awareness, improving the cultural responsiveness of services, and addressing social determinants of health, such as poverty and intimate partner violence. Some specific strategies included tailoring educational messages in consultation with patient advisory groups, engaging peer health workers, and addressing social challenges through referrals to community services. Clinicians also reported a need for greater racial/ethnic and linguistic diversity among health providers to increase non-White women’s comfort with seeking treatment.

Patients and clinicians also perceived room for *improvement in how clinicians discussed and managed perinatal depression.* Clinicians reported a need for more training on perinatal depression among OBGYN and mental health providers. Clinicians also viewed an opportunity to increase providers’ knowledge of cultural and socioeconomic factors and ways to tailor treatment recommendations.

Finally, patients and clinicians favored strategies to *integrate OBGYN care and mental health treatment*. Multiple participants recommended embedding a behavioral health clinician in the OBGYN clinic who could see the patient soon after a positive depression screen and establish treatment. This integrated approach increased convenience, avoided the stigma associated with a general mental health referral, and framed depression as a medical complication requiring treatment.

## Discussion

This qualitative study examined perinatal depression treatment engagement factors as observed by racially/ethnically diverse obstetric patients and by clinician experts in perinatal depression. Barriers across race/ethnicity included difficulty recognizing the severity of depressive symptoms, low understanding of treatment processes, and time constraints. Support from clinicians and families was critical for treatment engagement, and conversely, family disapproval or clinician missteps in establishing trust could derail patients’ help-seeking. Women across racial/ethnic groups described treatment-related stigma but characterized these beliefs distinctly within groups. Women from non-White backgrounds also faced more challenges posed by lack of social support, trauma history, and difficulty getting time off work for treatment. Participants noted that treatment would be facilitated by increasing outreach, convenience, community involvement, cultural inclusivity, and behavioral health integration.

Our findings on social stigma echoed past research reporting that women may avoid treatment due to cultural messages regarding the shame of mental illness and its incompatibility with idealized images of motherhood [20, 23, 24]. Fears of discrimination, e.g., in relation to immigration status or child custody, have also been reported elsewhere [35, 36]. Patients and clinicians in our study viewed cultural factors as relevant to understanding barriers to treatment engagement and that they could be overcome through trusting and collaborative patient-provider relationships. Establishing trust with clinicians may be especially challenging but also important for trauma survivors and others who lack support from their families to pursue treatment.

Clinician training and screening response protocols should be designed with cultural factors in mind, with messages emphasizing the commonness of perinatal depression across race/ethnicity and socioeconomics. Repeated and proactive conversations with at-risk patients may be necessary to help women overcome initial reluctance and to facilitate treatment initiation and engagement. Recently, the U.S. Preventive Services Task Force underscored the need for preventive care directed to those who are vulnerable to perinatal depression [37].

Some clinicians in our study viewed patients as unlikely to accept antidepressant medications for perinatal depression. Past research, including among non-White women, has suggested a reluctance to take medications for perinatal depression [17, 23, 26, 35, 38]. In the general population, non-White patients are less likely than White patients to use antidepressants in many settings [39–41]. In this study, we did not see clear preferences by race/ethnicity. Some patients did prefer pursuing psychotherapy before trying medications, but others saw medications as key to their recovery, and conferring fewer health risks than depression itself. Psychotherapy incorporating social support (as in perinatal depression groups) was seen as helpful and acceptable if presented to patients in a non-stigmatizing manner. Participants perceived an important role for OBGYN physicians in exploring concerns with patients and helping them weigh various options through a collaborative, patient-centered approach.

Lack of clinician knowledge or skill in managing perinatal depression or related cultural factors have been observed in past research [20, 23, 24]. Clinician education on perinatal depression and cultural factors, as well as greater integration and collaboration between OBGYN and mental health providers, may facilitate follow-through to treatment. Adaptation of clinical care to women’s time constraints, their socioeconomic challenges, and their quality of social support is necessary for devising a feasible treatment plan. Newer treatment modalities delivered virtually may be particularly well-suited to women during the perinatal period given unique scheduling challenges.

Study findings should be considered in the context of several limitations. Due to study population characteristics, findings may not generalize to women who have low English language proficiency, lack health insurance, or receive care in non-integrated healthcare systems. Given the small number of participants in each racial/ethnic subgroup, this study may not capture the full range of perspectives per group, including within subgroups based on language or country of origin. To provide additional cultural perspectives, we included interviews with clinicians treating ethnically diverse populations with perinatal depression. Our reliance on a depression screening measure to determine eligibility may have reduced study generalizability given that some women may not disclose depressive symptoms on a clinical questionnaire. This limitation may have been mitigated by the standardized screening protocol, which included repeated screening in the perinatal period using a validated measure [13]. Study findings should be confirmed in larger studies that allow for examination of cultural, linguistic, socioeconomic, and health system-related factors. Strengths of this study included the purposeful recruitment of women from distinct racial/ethnic backgrounds and pregnancy stages from across a large region, the use of telephone focus groups to increase representativeness and facilitate participant disclosure [31], and the focus on barriers other than mental health service access given KPNC’s comprehensive perinatal depression screening and treatment.

Prompt identification and treatment of perinatal depression should be viewed as essential components of obstetric care. Our findings suggest the importance of intervention and policy approaches effecting change at the patient, family, clinician, and community level. The recommendations offered by this study’s participants included multilevel educational initiatives, greater engagement of community resources with attention to social determinants of health; and changes in service provision to foster convenience and integration across medical specialties.

## Conclusions

Although perinatal depression screening has increased in recent years, many women who screen positive do not receive mental health treatment, with the lowest rates found for non-White women. In this qualitative study that included women across racial/ethnic groups with significant perinatal depressive symptoms and clinicians serving this population, we focused on treatment engagement barriers that persisted despite universal perinatal depression screening and well-defined treatment pathways in a large healthcare system. The many noted treatment barriers—e.g., awareness gaps (both patients’ and clinicians’), treatment inconvenience, and culturally specific social stigma—are likely to arise in diverse healthcare settings and to particularly hinder women with existing economic and social vulnerabilities. Integration of depression care into obstetrics services, such as through an embedded behavioral health provider, addressed multiple logistical and stigma-related barriers for pregnant and postpartum women with depression.

## Data Availability

The datasets used during the current study are available from the corresponding author upon reasonable request.

## Abbreviations

COM-B: Capability-Opportunity-Motivation-Behavior
COVID-19: Coronavirus Disease-2019
EHR: Electronic health record
KPNC: Kaiser Permanente Northern California
OBGYN: Obstetrics and gynecology
PHQ-9: Patient Health Questionnaire, 9-item
